# The reliability of WorkWell Systems Functional Capacity Evaluation: a systematic review

**DOI:** 10.1186/1471-2474-15-106

**Published:** 2014-03-27

**Authors:** Sebastian Bieniek, Matthias Bethge

**Affiliations:** 1 Department for Rehabilitation Medicine, Hannover Medical School, Carl-Neuberg-Straße 1, 30625 Hannover, Germany

**Keywords:** Functional capacity evaluation, Assessment, Reliability, Systematic review, WorkWell Systems, Isernhagen

## Abstract

**Background:**

Functional capacity evaluation (FCE) determines a person’s ability to perform work-related tasks and is a major component of the rehabilitation process. The WorkWell Systems (WWS) FCE (formerly known as Isernhagen Work Systems FCE) is currently the most commonly used FCE tool in German rehabilitation centres. Our systematic review investigated the inter-rater, intra-rater and test-retest reliability of the WWS FCE.

**Methods:**

We performed a systematic literature search of studies on the reliability of the WWS FCE and extracted item-specific measures of inter-rater, intra-rater and test-retest reliability from the identified studies. Intraclass correlation coefficients ≥ 0.75, percentages of agreement ≥ 80%, and kappa coefficients ≥ 0.60 were categorised as acceptable, otherwise they were considered non-acceptable. The extracted values were summarised for the five performance categories of the WWS FCE, and the results were classified as either consistent or inconsistent.

**Results:**

From 11 identified studies, 150 item-specific reliability measures were extracted. 89% of the extracted inter-rater reliability measures, all of the intra-rater reliability measures and 96% of the test-retest reliability measures of the weight handling and strength tests had an acceptable level of reliability, compared to only 67% of the test-retest reliability measures of the posture/mobility tests and 56% of the test-retest reliability measures of the locomotion tests. Both of the extracted test-retest reliability measures of the balance test were acceptable.

**Conclusions:**

Weight handling and strength tests were found to have consistently acceptable reliability. Further research is needed to explore the reliability of the other tests as inconsistent findings or a lack of data prevented definitive conclusions.

## Background

To prevent health-related early retirement and to promote participation in working life, social security agencies in many Western countries provide rehabilitation services for persons with limited work ability if they are expected to return to work after rehabilitation. In Germany, two types of rehabilitation services are provided to different groups of working aged persons according to the degree of limitation of functioning: general medical rehabilitation and work-related medical rehabilitation (WMR). The objectives of both rehabilitation strategies are to achieve long-term improvements in work capacity and reduce the risk of disability pensions [1,2]. Patients with severe limitations of work-related functioning receive WMR [3-7], which comprises multimodal rehabilitation programs that follow the principles of functional restoration [8] and work hardening [9].

The recently published German WMR guideline [10] recommends a job-specific mini functional capacity evaluation (FCE) at the beginning of work-related medical rehabilitation to objectively measure the patients’ ability to perform functional work activities. These data supplement the medical history and provide information on treatment requirements, return to work or functional capacity-based workplace configuration. The WorkWell Systems (WWS) FCE (formerly known as Isernhagen Work Systems FCE) [11] is currently the most commonly used FCE tool in German rehabilitation centres. It was developed by Susan Isernhagen in the 1980s as a systematic method to objectively assess a subject’s ability to perform work-related tasks. The complete test battery consists of 29 items related to five performance categories (weight handling and strength, posture and mobility, locomotion, balance, hand coordination) (Table 1). For the six weight handling tests, the tasks must be repeatedly performed whilst the load is gradually increased to the level of maximal safe performance. Usually, this is done in six steps. In each step, the therapist assigns the subject one of four effort levels (light, moderate, heavy and maximal safe performance), which are defined by standardised observation criteria. Other tests are characterised by criteria or ceilings. Tests with a criterion are fulfilled if a specified criterion is met, e.g., a person is able or unable to push a weighted cart over a distance of 20 m safely. Tests with a ceiling are fulfilled if the ceiling is reached, which means that a subject has met the defined maximal time of performance, e.g., the working overhead test is terminated if a person has reached 15 min even though the person might have not performed to her or his maximal ability [12]. Additionally, there are some other tests without criteria or ceilings which directly assess the safe maximum or average performance capacity (i.e. pushing or pulling static, shuttle walk). Additional information on materials, training, certificates, and costs of the WWS FCE have been reported by Genovese and Galper [13].

**Table 1:** Workwell Systems Functional Capacity Evaluation subtests

**Performance category**	**WWS FCE subtests**
Weight handling and strength	Lifting low
	Lifting high
	Short carry
	Long carry
	Long carry right-handed
	Long carry left-handed
	Pushing static
	Pulling static
	Pushing dynamic
	Pulling dynamic
	Grip strength right
	Grip strength left
Posture and mobility	Overhead work
	Forward bent standing
	Forward bent sitting
	Kneeling
	Crawling
	Crouching
	Dynamic squatting
	Repetitive rotation standing right/left
	Repetitive rotation sitting right/left
	Sitting tolerance
	Standing tolerance
Locomotion	Walking
	Stair climbing
	Ladder climbing
Balance	Balance
Hand coordination	Hand coordination right
	Hand coordination left

WWS FCE, WorkWell Systems Functional Capacity Evaluation.

Despite increasing use of the WWS FCE in rehabilitation, its inter-rater, intra-rater and test-retest reliability have been critically discussed in the scientific literature [14-16]. Inter-rater reliability is the consistency of measures or scores by different examiners on the same phenomenon, whereas intra-rater reliability describes the consistency between repeated assessments, assuming that the characteristic of interest does not change over time. In this review, inter-rater and intra-rater reliability refer to the effort level or the observation of safe performance. This is a major concern in the first six weight handling tests in Table 1 because the performance of these has to be judged by the evaluator whilst using standardised observation criteria. Test-retest reliability in this review refers to the consistency of the tested capacity. This capacity of the individual under evaluation can be expressed as a continuous measure, e.g. in kg or N, or as the achievement or non-achievement of a criterion or ceiling.

Three previous reviews have examined the reliability of the WWS FCE. In 2004, Gouttebarge and colleagues [15] found that the WWS FCE has moderate to good inter-rater reliability but could not reach a definitive conclusion about its intra-rater reliability due to methodological shortcomings of the identified studies. In 1999, Innes and Straker [14] also rated its inter-rater reliability as acceptable but had doubts about the methodology of intra-rater reliability studies. In a more recent paper, Innes [16] concluded from a narrative summary that most of the available studies have reported acceptable inter-rater, intra-rater and test-retest reliability. Since then, new studies on the reliability of the WWS FCE have been published which permit a more differentiated view of the reliability of single subtests. Therefore, we conducted a systematic literature review to summarise the existing study results on the inter-rater, intra-rater and test-retest reliability of the WWS FCE. We used the PRISMA statement as a guide for transparent reporting of our findings [17]. We did not register our study protocol.

## Methods

### Inclusion criteria

Studies focusing on inter-rater, intra-rater or test-retest reliability of the WWS FCE were considered for inclusion. All studies providing quantitative data on the inter-rater, intra-rater or test-retest reliability in adults (at least 18 years of age) were included.

### Systematic search strategy

A systematic literature search was performed using the PubMed, Scopus and Web of Science electronic databases in May 2012. Only studies published in English or German since 1 January 1990 were included. An update of this search was performed in February 2014.

Using a five-step search strategy, we generated keywords by decomposing the term “functional capacity evaluation” into single components. A synonym was chosen for each component. Components and synonyms were connected by “OR” (steps 1 to 3), and these phrases were concatenated by “AND” (step 4). Finally, the resulting phrase was connected with “reliability” by “AND” (step 5) (Table 2).

**Table 2:** Electronic search strategy

**Step**	**Phrase**
#1	*Functional* OR *physical*
#2	*Capacity* OR *performance*
#3	*Evaluation* OR *assessment*
#4	#1 AND #2 AND #3
#5	#4 AND reliability

Furthermore, references were checked from three previously published systematic reviews on the reliability and validity of FCE [14-16] and from the reference lists of the identified studies. We also screened all abstract volumes of the German Rehabilitation Research Congress for potentially relevant articles.

### Study selection

The first author and another scientific colleague independently screened the titles of the identified references for potential relevance. All titles that were identified as potentially relevant by at least one researcher were checked by the first author for relevance based on their abstracts. Then, the first author applied the inclusion criteria to the full text of the selected articles.

### Assessment of methodological quality

The methodological quality of the included studies was assessed using the COSMIN checklist [18]. This checklist is increasingly used in systematic reviews of studies on measurement properties like reliability. The original COSMIN checklist for reliability studies consists of 14 items. Two items that refer to ordinal rating scores were omitted. One item that refers to the number of measurements which were available was omitted as inter-rater, intra-rater and test-retest reliability studies always need at least two measurements. Three items of the COSMIN checklist which refer to the time interval between measurements were only applied to intra-rater and test-retest reliability studies. If a paper reported on more than one type of reliability, we appraised the methodological quality of these study arms separately. The item that refers to the time interval in case of repeated measurements was specified according to Gouttebarge and colleagues [15]. Time intervals from 3 to 21 days were rated as appropriate.

We used the COSMIN checklist version with four response options [18]. This version defines excellent, good, fair and poor levels for each item of the checklist. Table 3 shows the modified version, which we used for our quality assessment. For some items, only two or three levels are defined, e.g. there are only two levels defined for the reporting of kappa in the case of dichotomous or nominal scores (kappa calculated is rated excellent vs. only percentage agreement calculated is rated poor). As recommended, the overall methodological quality of a study was obtained by taking the lowest rating of all of the items which were assessed, i.e. the worst score determined the study’s overall methodological quality. Consequently, if one item was scored as poor, the methodological quality of a reliability study was rated as poor. As recommended, we do not present quality ratings on an item level, but only the overall quality rating and the reason for that rating, i.e. the lowest scored items. Both authors independently scored all studies; in cases of disagreement on study items, consensus was reached by discussion.

**Table 3:** Modified COSMIN checklist for methodological quality assessment

	**Requirements**	**Excellent**	**Good**	**Fair**	**Poor**
1	Was the percentage of missing items given?	Percentage of missing items described	Percentage of missing items not described	-	-
2	Was there a description of how missing items were handled?	Described how missing items were handled	Not described but it can be deduced how missing items were handled	Not clear how missing items were handled	-
3	Was the sample size included in the analysis adequate?	Adequate sample size (≥ 100)	Good sample size (50–99)	Moderate sample size (30–49)	Small sample size (< 30)
4	Were the administrations independent?	Independent measurements	Assumable that the measurements were independent	Doubtful whether the measurements were independent	Measurements not independent
5	Was the time interval stated?	Time interval stated	-	Time interval not stated	-
6	Were patients stable in interim period on the construct to be measured?	Patients were stable (evidence provided)	Assumable that patients were stable	Unclear whether patients were stable	Patients were not stable
7	Was the time interval appropriate?	Time interval between test-retest ranges from 3 to 21 days	-	Doubtful whether time interval was appropriate	Time interval between test-retest is less than 3 or more than 21 days
8	Were the tests conditions similar for both measurements? e.g., type of administration, environment, and instructions	Test conditions were similar (evidence provided)	Assumable that test conditions were similar	Unclear whether test conditions were similar	Test conditions were not similar
9	Were there any important flaws in the design or methods of the study?	No other important methodological flaws in the design or execution of the study	-	Other minor methodological flaws in the design or execution of the study	Other important methodological flaws in the design or execution of the study
10	For continuous scores: Was ICC calculated?	ICC calculated and model or formula of the ICC is described	ICC calculated but model or formula of the ICC not described. Pearson or Spearman correlation coefficient calculated with evidence provided that no systematic change has occurred	Pearson or Spearman correlation coefficient calculated without evidence provided that no systematic change has occurred or with evidence that systematic change has occurred	No ICC or Pearson or Spearman correlations calculated
11	For dichotomous/ nominal/ordinal scores: Was kappa calculated?	Kappa calculated	-	-	Only percentage agreement calculated

ICC, intraclass correlation coefficient. Items and definitions of quality levels are according to Terwee et al. [18]. Items 5, 6, and 7 were only applied on intra-rater and test-retest reliability studies. Specification of appropriate time intervals follows Gouttebarge et al. [15].

### Synthesis of primary studies

We extracted intraclass correlation coefficients (ICC) as reliability statistics for continuous measures and percentages of agreement (POA) and kappa coefficients (κ) for binary measures. Item-specific reliability measures were rated as acceptable if ICC ≥ 0.75, POA ≥ 80%, or κ ≥ 0.60, otherwise they were considered non-acceptable [14,15]. Data were extracted by the first author in Microsoft Excel sheets separately for inter-rater, intra-rater and test-reliability and the three reliability measures. Each of the sheets comprised a matrix of the included studies by the 29 items. All of the extracted values were checked by the second author. Disagreement was dissolved by discussion. The extracted values were pooled for five performance categories: strength and weight handling, posture and mobility, locomotion, balance, and hand coordination. The consistency of the results was rated on two levels (consistent vs. inconsistent). We rated the results for one performance category to be consistent if at least 75% of the extracted values of agreement coincided; otherwise, inconsistency was stated.

## Results

### Literature search

Our literature search yielded 5403 hits (PubMed: n = 1787; Scopus: n = 1730; Web of Science: n = 1886). The inter-sectionality of the primary references identified through the respective databases was poor; the highest agreement was between PubMed and Scopus (26%), followed by PubMed and Web of Science (19%). The inter-sectionality between Scopus and Web of Science was only 16%.

**Figure 1:**
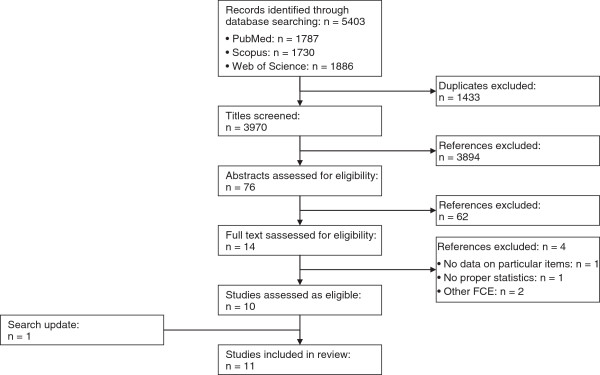
Flowchart of study selection.

After eliminating duplicates (exclusion: n = 1433), two researchers independently screened the remaining 3970 references by applying the inclusion criteria to the titles. This led to the exclusion of 3894 articles. Titles considered potentially relevant by at least one researcher were again reviewed for relevance of the abstract by the first author (n = 76). Among these articles, 62 did not meet the inclusion criteria and were therefore excluded. The first author applied the inclusion criteria to the full texts of the remaining 14 articles [12,19-31]. Four references were excluded: one [21] because it did not report any data on the reliability of specific items, two because the investigators tested FCE systems other than the WWS FCE [27,31], and one because relevant statistics were not reported [22]. Our search update in February 2014 identified two potentially relevant studies [32,33]. We included one of the studies in our review [32]. We excluded the other study as the low number of observed performances (1 to 4) per item was considered to be inadequate for a reasonable item-specific analysis [18]. Finally, 11 articles [12,19,20,23-26,28-30,32] were deemed eligible for inclusion in our review. Figure 1 shows a flowchart of the study selection process.

No additional references were identified by checking the reviews by Innes and Straker [14], Gouttebarge and colleagues [15] and Innes [16] or by screening the reference lists of the included papers and searching in the abstract volumes of the German Rehabilitation Research Congress. We identified nine additional studies compared to the review by Innes and Straker [14], six additional studies compared to the review by Gouttebarge and colleagues [15], and four additional studies compared to the narrative review by Innes [16]. Table 4 shows the main characteristics of the 11 studies included in the review.

**Table 4:** Characteristics of the included studies

							**Inter-rater reliability**	**Intra-rater reliability**	**Test-retest reliability**			
**Authors**	**Population**	**Health status of subjects**	**Effort levels assessed**	**Type of monitoring**	**Expertise of observers**	**Range of included items**	**Weight handling and strength**	**Weight handling and strength**	**Weight handling and strength**	**Posture and mobility**	**Locomotion**	**Balance**
Smith [19]	*n*: 21 subjects (126 taped procedures); *Age*: 40 years (22–61); *Sex*: 16 M, 5 F; *r*: 5 raters; *Country*: USA	Low back pain	Maximal safe performance	Video monitoring	Trained	Lifting low	POA: 81-82%; κ: 0.62-0.64	POA: 87%; κ: 0.73				
Gardener and McKenna [20]	*n*: 30 subjects (144 taped procedures); *Age:* 31 years (20–30); *Sex:* 10 M, 20 F; *r*: 5 raters; *Country*: Australia	Unclear	Maximal safe performance	Video monitoring	Trained	Lifting low	POA: 86-94%; κ: 0.56-0.82					
Gross and Battie [23]	*n*: 28 subjects; *Age:* 41 years (23–62); *Sex:* 20 M, 8 F; *r*: 5 raters; *Country*: Canada	Low back pain	Maximal safe performance	Direct supervision	Trained	Lifting low, lifting high, short carry two-handed, long carry two-handed, long carry left-handed, long carry right-handed	ICC: 0.95-0.98		ICC: 0.78-0.94			
Reneman et al. [24]	*n*: 4 subjects (104 taped procedures); *Age:* 20–30 years; *Sex:* 2 M, 2 F; *r*: 5 raters; *Country*: Netherland	Healthy	Light, moderate, heavy and maximal safe performance	Video monitoring	Trained	Lifting low, lifting high, short carry two-handed, long carry two-handed, long carry left-handed, long carry right-handed	POA: 87-96%	POA: 93-97%				
Reneman et al. [25]	*n*: 50 subjects; *Age*: 38.8 (28–52); *Sex*: 39 M, 11 F; *Country*: Netherland	Chronic low back pain	Maximal safe performance	Direct supervision	Trained	Lifting low, lifting high, short carry two-handed			ICC: 0.77-0.87			
Brouwer et al. [12]	*n*: 30 subjects; *Age*: 40 years; *Sex*: 24 M, 6 F; *Country*: Netherland	Chronic low back pain	Maximal safe performance	Direct supervision	Trained	Complete WWS protocol except for grip strength and hand coordination			ICC: 0.75-0.87; POA: 100%	ICC: 0.36-0.96; POA: 78-100%; κ: 0.51-1.00	ICC: 0.84; POA: 78-85%; κ: 0.25-0.56	POA: 96%
Reneman et al. [26]	*n*: 26 subjects; *Age*: 34.9 years; *Sex*: 14 M, 12 F; *Country*: Netherland	Healthy	Maximal safe performance	Direct supervision	Trained	Complete WWS protocol except for grip strength and hand coordination			ICC: 0.68-0.98; POA: 100%	ICC: 0.54-0.93; POA: 79-100%; κ: 0.57-1.00	ICC: 0.64; POA: 85-100%; κ: 0.69	POA: 100%
Reneman et al. [28]	*n*: 15 subjects (71 taped procedures); *Age*: 29.5 years (18–53); *Sex*: 7 M, 8 F; *r*: 9 raters; *Country*: Netherland	Healthy	Light, moderate, heavy and maximal safe performance	Video monitoring	Trained	Lifting low	κ: 0.58					
	*n*: 16 subjects (63 taped procedures); *Age*: 39.6 years (27–50); *Sex*: 12 M, 4 F; *r*: 9 raters; *Country*:Netherland	Chronic low back pain	Light, moderate, heavy and maximal safe performance	Video monitoring	Trained	Lifting low	κ: 0.50					
Soer et al. [29]	*n*: 33 subjects; *Age*: 29.2 years; *Sex*: 14 M, 19 F; *Country*: Netherland	Healthy	Maximal safe performance	Direct supervision	Trained	Lifting high, grip strength right, grip strength left, overhead work			ICC: 0.86-0.92	ICC: 0.90		
van Ittersum et al. [30]	*n*: 79 subjects; *Age*: 56.6 years (45–65); *Sex*: 15% M, 85% F; *Country*: Netherland	Osteoarthritis (hip and/or knee)	Maximal safe performance	Direct supervision	Trained	Lifting low, lifting high, short carry two-handed			ICC: 0.75-0.88			
Trippolini et al. [32]	*n*: 32 subjects; *Age*: 36.9 years (18–65); *Sex*: 21 M, 11 F; *Country*: Switzerland	Whiplash-associated disorders	Maximal safe performance	Direct supervision	Trained	Lifting low, lifting high, short carry two-handed, long carry left-handed, long carry right-handed, grip strength right, grip strength left, overhead work			ICC: 0.66-0.96	ICC: 0.83		

n, number of subjects; M, male; F, female; r, number of raters (only for studies on inter-rater reliability); ICC, intraclass correlation coefficient; POA, percentage of agreement; κ, kappa coefficient.

Five of the studies analysed subjects with musculoskeletal disorders [12,19,23,25,30]. One study assessed healthy persons as well as patients with chronic musculoskeletal pain [28]. One study focused on persons who had suffered whiplash injuries [32]. Three studies [24,26,29] evaluated healthy subjects; in one study the health status of the subjects was unclear [20]. Only one study [24] distinguished between all four effort levels (light, moderate, heavy, maximal), while the others focused on submaximal vs. safe maximal performance. All assessments were performed by trained observers. The items were rated under direct supervision in seven studies [12,23,25,26,29,30,32], and by video monitoring in four studies [19,20,24,28]. Only two of the studies assessed the complete battery of items except for grip strength and hand coordination [12,26]. Of the remaining nine studies, one evaluated eight items [32], two tested six items [23,24], one evaluated four items [29], two tested three items [25,30], and three assessed only one item [19,20,28].

### Methodological quality assessment

Both authors independently assessed the methodological quality by type of reliability of all 11 studies; overall ratings were completely consistent. Methodological quality of inter-rater reliability studies was rated as good three times [19,20,28] and otherwise as poor [23,24]. Methodological quality for intra-rater or test-retest reliability was rated as fair two times [29,32] and otherwise as poor [12,19,23-26,30] (Table 5). The major methodological limitations were insufficient sample size and inappropriate time intervals in the case of studies on intra-rater or test-retest reliability.

**Table 5:** Methodological quality assessment

**Authors**	**Methodological quality by type of reliability**			
	**Inter-rater**		**Intra-rater or test-retest**	
	**Methodological quality**	**Worst scores**	**Methodological quality**	**Worst scores**
Smith [19]	Good	4) Assumable that the measurements were independent	Poor	7) Time interval not appropriate
Gardener and McKenna [20]	Good			8) Assumable that test conditions were similar
Gross and Battie [23]	Poor	3) Small sample size (< 30)	Poor	3) Small sample size (< 30)
				7) Time interval not appropriate
Reneman et al. [24]	Poor	11) Only percentage agreement calculated	Poor	7) Time interval not appropriate;
				11) Only percentage agreement calculated
Reneman et al. [25]			Poor	7) Time interval not appropriate
Brouwer et al. [12]			Poor	6) Patients were not stable
Reneman et al. [26]			Poor	3) Small sample size (< 30)
Reneman et al. [28]^a^	Good			3) Good sample size (50–99)^c^
Reneman et al. [28]^b^	Good			3) Good sample size (50–99)^c^
Soer et al. [29]			Fair	3) Moderate sample size (30–49)
van Ittersum et al. [30]			Poor	7) Time interval not appropriate
Trippolini et al. [32]			Fair	3) Moderate sample size (30–49)

^a^Healthy subjects; ^b^Patients with chronic low back pain; ^c^Number of taped observations were appraised.

### Inter-rater reliability

We extracted 28 inter-rater reliability measures for the various tests of weight handling and strength. None could be identified for the other tests.

#### ***Weight handling and strength***

The inter-rater reliability of lifting low assessment was evaluated in five studies [19,20,23,24,28]. Gross et al. [23] reported acceptable intraclass correlation (ICC = 0.98) for maximal effort determinations by two independent raters. Three studies showed an acceptable percentage of agreement (81% ≤ POA ≤ 90%), i.e. consistency between performance assessments by different observers [19,20,24]. Kappa coefficients for inter-rater reliability were not consistently acceptable [19,20,26,28]. Results on the reliability of five other subtests of weight handling and strength (lifting high, short carrying, long carry two-handed, long carry right-handed and left-handed) were also available. Gross et al. [23] reported a high intraclass correlation for these items (0.95 ≤ ICC ≤ 0.96). Percentages of agreement of 87% and higher were reported by Reneman et al. [24].

#### ***Synthesis of results***

Six intraclass correlations, 16 percentages of agreement and 3 of 6 kappa coefficients showed an acceptable level of inter-rater reliability. Thus, 25 out of 28 measures (89%) reported values which indicated that the inter-rater reliability of lifting and carrying assessments was acceptable. No inter-rater reliability data for the other items of the WWS FCE could be found.

### Intra-rater reliability

We extracted eight intra-rater reliability statistics for the weight handling and strength tests.

#### ***Weight handling and strength***

Two studies evaluated the intra-rater reliability of lifting low [19,24]; both showed an acceptable percentage of agreement between repeated measurements (78% ≤ POA ≤ 94%) and one [19] showed an acceptable kappa coefficient (κ = 0.73). One study showed an acceptable percentage of agreement for lifting high, short and long carry both-handed, as well as long carry right- and left-handed (93% ≤ POA ≤ 97%) [24].

#### ***Synthesis of results***

The extracted data indicated that the intra-rater reliability of the subtests of strength and weight handling was acceptable, as determined by the seven extracted percentages of agreement and one kappa coefficient. Thus, the eight reported intra-rater reliability measures for the weight handling and strength items were consistently acceptable.

### Test-retest reliability

We extracted 48 test-retest reliability statistics for the weight handling and strength tests, 55 for the posture/mobility tests, nine for the locomotion tests, and two for the balance test. No studies examining the test-retest reliability of hand coordination assessments could be identified.

#### ***Weight handling and strength***

Six studies [12,23,25,26,30,32] showed acceptable intraclass correlation for repeated measurements of lifting low (0.78 ≤ ICC ≤ 0.95). Six studies showed acceptable intraclass correlation for lifting high (0.75 ≤ ICC ≤ 0.92) [12,23,25,26,29,30], while one did not (ICC = 0.66) [32]. Six studies showed acceptable intraclass correlation for short carry both-handed assessments (0.77 ≤ ICC ≤ 0.96) [12,23,25,26,30,32]. Intraclass correlation between long carry both-handed (0.81 ≤ ICC ≤ 0.90) [12,23,26], long carry left-handed (0.81 ≤ ICC ≤ 0.91) [12,23,26,32] and long carry right-handed assessments (0.81 ≤ ICC ≤ 0.98) [12,23,26,32] was also acceptable.

We also found acceptable intraclass correlation between pulling static scores (0.78 ≤ ICC ≤ 0.89) [12,26]. For pushing static scores, the intraclass correlation was acceptable in one study (ICC = 0.75) [12], and not acceptable in another study (ICC = 0.68) [26]. Additionally, for pulling and pushing dynamic, all results for percentages of agreement were acceptable [12,26]. Two of the identified studies assessed the test-retest reliability of the grip strength test to be consistently acceptable (right: 0.86 ≤ ICC ≤ 0.92; left: 0.88 ≤ ICC ≤ 0.89) [29,32].

#### ***Synthesis of results***

The extracted data indicated that the test-retest reliability of the subtests of strength and weight handling was acceptable, as determined based on 42 of 44 (95%) intraclass correlation coefficients and all four of the extracted percentages of agreement. In total, 46 of 48 (96%) reported values were acceptable. Thus, the test-retest reliability for the weight handling and strength items was consistently acceptable.

#### ***Posture and mobility***

##### 

**Overhead work** Two studies reported unacceptable intraclass correlations for working overhead assessments (0.36 ≤ ICC ≤ 0.58) [12,26], but two further studies assessed acceptable intraclass correlation (0.83 ≤ ICC ≤ 0.90) [29,32]. Percentages of agreement were acceptable in two studies (96%) [12,26], and one of these studies reported an acceptable kappa coefficient (κ = 0.78) [26].

##### 

**Forward bend standing and sitting** The reliability of the two subtests of forward bent posture was quiet variable. For forward bend standing, two studies [12,26] showed acceptable intraclass correlations (0.93 ≤ ICC ≤ 0.96) and percentages of agreement (POA = 100%) [12,26], and one [26] reported an acceptable kappa coefficient (κ = 1.00). For forward bend sitting, however, these studies reported unacceptable intraclass correlation (ICC = 0.72) [12] and both acceptable and unacceptable percentages of agreement (79% ≤ POA ≤ 89%) and kappa coefficients (0.57 ≤ κ ≤ 0.60) [12,26].

##### 

**Kneeling, crawling, crouching, dynamic squatting** Percentages of agreement (78% ≤ POA ≤ 96%) and kappa coefficients (0.57 ≤ κ ≤ 0.65) for kneeling assessments were determined as acceptable in one study [11] and unacceptable in another [10]. Acceptable percentages of agreement and kappa coefficients were reported for crouching and crawling [12,26]. Acceptable percentages of agreement (96% ≤ POA ≤ 100%) and, in one case, an acceptable kappa coefficient (κ = 0.91) were reported for dynamic squatting [12,26]. However, intraclass correlations were quiet variable (0.54 ≤ ICC ≤ 0.82) [12,26].

##### 

**Repetitive rotation standing and sitting** Two studies reported unacceptable intraclass correlations for repetitive rotation standing and sitting of left and right side [12,26]. According to Brouwer et al. [12], kappa coefficients for repetitive rotation standing were unacceptable (left: κ = 0.58; right: κ = 0.51), but those for repetitive rotation sitting were acceptable (left: κ = 0.78; right: κ = 0.87) [12]. Percentages of agreement were acceptable for all items in both studies (85% ≤ POA ≤ 100%) [12,26].

##### 

**Sitting and standing tolerance** The only study examining sitting and standing tolerance reported acceptable percentages of agreement (93% ≤ POA ≤ 96%) [12].

##### 

**Synthesis of results** Only five of 17 (29%) intraclass correlation coefficients compared to 22 of 24 (92%) percentages of agreement and 10 of 14 (71%) kappa coefficients reported for the analysed posture and mobility subtests were determined to be acceptable. Overall, only 37 of 55 (67%) reported values were acceptable. Accordingly, the results of the identified studies are considered inconsistent.

##### 

**Locomotion** One of two studies [12] reported an acceptable intraclass correlation coefficient (ICC = 0.84) for walking assessments, whereas the other did not (ICC = 0.64) [26]. The reported percentages of agreement (78% ≤ POA ≤ 85%) and kappa coefficients (0.56 ≤ κ ≤ 0.69) for stair climbing were acceptable in one [26] and unacceptable in another study [12]. Regarding ladder climbing assessments, the percentage of agreement (85% ≤ POA ≤ 100%) was acceptable in both studies [12,26]; the extracted kappa coefficient was not at an acceptable level (κ = 0.25) [12].

##### 

**Synthesis of results** Three of the four (75%) percentages of agreement, but only one of three (33%) kappa coefficients and one of two (50%) intraclass correlation coefficients showed acceptable intra-rater reliability for the locomotion subtests. Overall, only five of nine (56%) reported values demonstrated acceptable test-retest reliability. Therefore, the results of the existing studies are considered inconsistent.

##### 

**Balance** Two studies reported acceptable agreement (POA = 96%) between balance assessments [12,26].

### 

**Overall reliability** Table 6 summarises all of the extracted reliability statistics. In total, 25 of 28 (89%) inter-rater reliability measures, all eight intra-rater measures, and 90 of 114 (79%) test-retest reliability measures were found to be at an acceptable level. Overall, 123 of 150 (82%) reliability statistics were at an acceptable level. Most of the extracted reliability statistics came from poor quality studies according to the COSMIN criteria [18] (poor: 128 reliability measures; fair to good: 22 reliability measures). There were no serious differences in reliability statistics between poor and fair to good quality studies. Reliability statistics were acceptable for the extracted intraclass correlation coefficients (54 of 69; 78%) and percentages of agreement (54 of 57; 95%) but not for the extracted kappa scores (15 of 24; 63%).

**Table 6:** Overall synthesis of reliability statistics

	**All studies**			**Level of methodological quality**					
				**Poor**			**Fair to good**		
	**Acceptable**	**Total**	**% Acceptable**	**Acceptable**	**Total**	**% Acceptable**	**Acceptable**	**Total**	**% Acceptable**
*Inter-rater reliability*									
Weight-handling and strength									
ICC	6	6	100.0%	6	6	100.0%			
κ	3	6	50.0%				3	6	50.0%
POA	16	16	100.0%	12	12	100.0%	4	4	100.0%
Total	25	28	89.3%	18	18	100.0%	7	10	70.0%
*Intra-rater reliability*									
Weight-handling and strength									
κ	1	1	100.0%	1	1	100.0%			
POA	7	7	100.0%	7	7	100.0%			
Total	8	8	100.0%	8	8	100.0%			
*Test-retest reliability*									
Weight-handling and strength									
ICC	42	44	95.5%	33	34	97.1%	9	10	90.0%
POA	4	4	100.0%	4	4	100.0%			
Total	46	48	95.8%	37	38	97.4%	9	10	90.0%
Posture and mobility									
ICC	5	17	29.4%	3	15	20.0%	2	2	100.0%
κ	10	14	71.4%	10	14	71.4%			
POA	22	24	91.7%	22	24	91.7%			
Total	37	55	67.3%	35	53	66.0%	2	2	100.0%
Locomotion									
ICC	1	2	50.0%	1	2	50.0%			
κ	1	3	33.3%	1	3	33.3%			
POA	3	4	75.0%	3	4	75.0%			
Total	5	9	55.6%	5	9	55.6%			
*Balance*									
POA	2	2	100.0%	2	2	100.0%			
Total	2	2	100.0%	2	2	100.0%			
Total test-retest reliability	90	114	78.9%	79	102	77.5%	11	12	91.7%
*Overall reliability statistics*	123	150	82.0%	105	128	82.0%	18	22	81.8%

ICC, intraclass correlation coefficient; κ, kappa coefficient; POA, percentage of agreement.

## Discussion

Five studies [19,20,23,24,28] examined the inter-rater reliability of one or more weight handling subtests. Overall, 25 of 28 (89%) reported values were at an acceptable level. Consequently, the reliability measures were consistently acceptable [19,20,23,24,28]. Intra-rater reliability was also consistently acceptable for these tests, as indicated by eight reliability statistics from two studies [19,24]. Additionally, 46 of 48 (96%) reliability statistics that were extracted from seven test-retest reliability studies [12,23,25,26,29,30,32] were acceptable, consistently suggesting an acceptable test-retest reliability of the weight handling tests. However, the results of the posture/mobility and locomotion subtests were inconsistent. The reliability of the balance subtest was consistent, but there were only two studies available. The reliability of the hand coordination subtest has not been analysed in any study to date.

The methodological quality of the included studies was appraised using the COSMIN checklist [18]. Our objective was to determine the risk of bias during the reliability assessment. Some of the studies were originally performed to investigate different research questions, e.g. if a second day of testing is needed as recommended in the original WWS FCE protocol [25]. In such a study, a time interval of one day was certainly appropriate to answer the original research question. However, this short time interval will likely introduce bias in the determination of the test-retest reliability of a procedure. Accordingly, the result of our quality assessment of this study is a consequence of our decision to include this study in our review. We therefore emphasize, that our appraisal of the methodological quality is limited to the scope of this review and should not be understood as an appraisal of current FCE research. Moreover, though the COSMIN checklist is increasingly used to appraise the methodological quality of reliability studies, the application of some items for the purpose of this review is debatable. As previously stated by Terwee et al. [18], the appraisal of the sample size given by the COSMIN authors is only a rule of thumb. Larger samples increase the precision of estimates, and therefore the results of studies using larger samples have a lower risk of bias. Nonetheless, small samples might also produce precise estimates and small confidence intervals, especially if homogenous samples were recruited. In this case, however, this could indicate a strong risk of selection bias. What is more important is that our separate analyses of poor and fair to good quality studies have not indicated serious differences in the reliability statistics. We therefore have concluded that the identified shortcomings of the studies have not effectively biased their findings, and decided to base our summary and final interpretation of the data equally on all identified studies.

Another important finding of our analysis is that the extracted percentages of agreement are more favourable than the extracted kappa scores. If the reaching of ceilings or criteria results in very differing proportions, e.g. when a test is too easy for the population under observation, high percentages of agreement (due to a large proportion of participants reaching the ceiling) will nevertheless result in low kappa scores. Similarly, kappa scores of inter-rater reliability will be low if proportions of maximal and submaximal test performances differ markedly. From a statistical point of view, high percentages of agreement but low kappa scores might indicate shortcomings in the study design that could be easily overcome, e.g. by the inclusion of a stratified sample that includes equal proportions of maximal and submaximal test performances.

Although FCE is of increasing importance in German rehabilitation settings, especially in work-related medical rehabilitation, and the WWS FCE is the most common assessment currently used, we could not identify any relevant German study. Though we do not assume major differences across countries, this certainly indicates a major lack of performance-related diagnostic rehabilitation research in Germany compared to the Netherlands, Canada and the United States. One reason is that the academic development of physiotherapy is in its infancy in Germany. As high-quality FCE research can contribute to improve the quality of rehabilitation services [34], we see this field as a major challenge for German rehabilitation research.

The WWS FCE was designed to assess the individual capacity to perform typical work-related tasks; it is used in rehabilitation, job placement decisions, and disability benefit evaluation. Thus, test results have significant consequences for patients, rehabilitation providers and insurance agencies. To rely on these results, reliability as well as validity needs to be demonstrated. However, only two of the eleven studies included examined almost the entire WWS FCE protocol [12,26]. The other nine studies mainly analysed weight handling and strength subtests, most frequently the lifting low task. Similarly, studies on the validity of the WWS FCE have focused on these procedures [35,36]. As these subtests achieve acceptable reliability and also appear to be predictive for time to return to work [35,36], shorter FCE protocols might be appropriate for several patients. For instance, Gross and colleagues [35] proposed a short-form FCE including lifting low, crouching and standing, which achieved comparable predictive ability like the complete WWS FCE. However, if complete assessment of a subject’s capability to work is required, the entire test protocol has to be performed, e.g. if an FCE supports a job placement decision or is performed as a prelude to a claim settlement. Therefore, further studies should be performed to evaluate the reliability of subtests that have been less frequently considered to date. Additional research is also needed as the results of posture/mobility and locomotion subtests are inconsistent. Interestingly, Soer et al. [29] have indicated that minor modifications of the original WWS FCE protocol, e.g. the use of cuff-weights in the case of overhead work, might improve the reliability of some other test procedures.

A critical discussion of our work must consider the following limitations of our review. Firstly, although we performed a systematic literature search, potentially relevant studies may have been overlooked. Our search terms may not have represented the complete range of relevant keywords. Moreover, our search was limited to articles published in English or German.

Secondly, we relied on three reliability statistics (intraclass correlation, kappa, percentage of agreement). We are aware that other statistics are also available. For the statistics used, however, references are available that could be used to categorize the extracted measures as indicating an appropriate or an inappropriate level of reliability (e.g. an ICC ≥ 0.75 is usually interpreted to indicate an appropriate reliability level) [14,15].

However, these limitations are accompanied by the following strengths.

Firstly, our comprehensive search strategy included three different electronic databases, and identified a set of new studies which were not included in previous reviews [14-16].

Secondly, we extracted item-specific reliability statistics and chose a more differentiated approach to data synthesis than previous reviews. Consequently, our analyses provide a comprehensive overview of the reliability of the WWS FCE. At the same time, we avoided the mistake of overgeneralising the acceptable reliability of some tests, e.g. lifting low, to the complete WWS FCE.

Thirdly, we assessed the methodological quality of the included studies according to the COSMIN checklist [18] and identified major limitations across studies that should be considered in future research, especially sample sizes that were too small and inappropriate time intervals in the case of intra-rater and test-retest reliability studies.

## Conclusions

Our analysis confirmed that the inter-rater, intra-rater and test-retest reliability of the strength and weight handling subtests of the WWS FCE are acceptable. The results for the other subtests (posture/mobility and locomotion) are inconsistent or provide insufficient data for definitive conclusions (balance, hand coordination). Further research with improved methodological quality is necessary to strengthen the scientific basis of the measurement properties of the WWS FCE.

## Abbreviations

FCE: Functional Capacity Evaluation; WWS: WorkWell Systems; WMR: Work-related medical rehabilitation; ICC: Intraclass correlation coefficients; POA: Percentages of agreement.

## Competing interests

The authors declare that they have neither financial nor non-financial competing interests.

## Authors’ contributions

SB and MB corporately developed the search strategy. SB screened the search results and identified eligible references for this review. Both authors determined the quality criteria and performed the data extraction. SB and MB contributed equally in interpretation and discussion of the results and in drafting the manuscript. Furthermore, SB and MB finally read and approved the manuscript for submitting. Both authors read and approved the final manuscript.

## Pre-publication history

The pre-publication history for this paper can be accessed here:

http://www.biomedcentral.com/1471-2474/15/106/prepub
